# Reading psychology and news communication strategies for affective computing

**DOI:** 10.3389/fpsyg.2022.989415

**Published:** 2022-09-02

**Authors:** Beichun Xu

**Affiliations:** College of Communication, Changchun Normal University, Changchun, China

**Keywords:** reading psychological mechanisms, affective computing, conditional random fields, news communication strategies, multi-angle

## Abstract

Reading psychology is an important basis for formulating news strategies. The purpose of this paper is to study how to analyze and study the psychological mechanism of reading and news communication strategies based on affective computing. It described the conditional random field. This paper put forward the problem of affective computing, which is based on affective computing technology. Then it expounded the concept of conditional random fields and related algorithms, and designed and analyzed cases of news communication strategies. Through the period from April 1, 2022 to June 1, 2022, the 300 short videos of news and information with the highest playback volume respectively released by Z software were used as research samples, and a multi-angle analysis was carried out. Among the 300 selected news information videos, 252 videos are original content, accounting for 84.00%, and the other short videos are from the self-media of the platform. At present, it is necessary to continuously develop intelligent products that meet the needs of users at different stages, and carry out personalized design in strict accordance with the living habits and natural conditions of different groups of people. Then continuously improving the experience of different groups of people using new media products, so that their reading interest can be more effectively stimulated.

## Introduction

With the development of the era of big data, data has become an integral part of people’s lives and work. In the latest infrastructure strategies, data-based technologies such as 5G, artificial intelligence, industrial internet and IoT are inseparable from data. Whether information is collected or disseminated, data plays a central role. In the future, any branch and department will have intersections and connections with data, and journalism will not be left out. Data journalism was created in this context. It aims to bring readers innovative news products that combine data and news from new perspectives. From the perspective of reading psychology, most people’s reading psychology shows a strong desire for knowledge, relatively slow reading speed, and uneven understanding of reading content. In order to achieve the effective dissemination of news, the media should pay attention to the psychological characteristics of different groups of people, understand the feelings and needs of different groups of people, and develop new multimedia products for different groups of people and make different news dissemination strategies. For example, meeting the reading needs of different groups of people in different ways. Continuously improving the acceptance of media products by different groups of people to effectively promote the effective dissemination of media news among different groups of people.

The psychological mechanism of reading is an important basis for formulating news strategies. Ht A investigated how car-related factors influence the establishment of bus use intentions in a psychological survey. The results of a 270-sample dataset investigating three types of daily trips in the Japanese context show a mediating effect in support of the overall car use factor (Hoang-Tung and Kubota, 2019). Miki reviewed the research on the temporal characteristics of sentence processing from both reading and experimental approaches. He Introduced the differences in reading performance between different reading modes (silent reading and oral reading) (Uetsuki et al., 2017). Qo’Ldoshev AR conducted a theoretical model analysis of the causes and consequences of left-handed development, briefly considering the psychological characteristics of left-handed children (Qo’Ldoshev, 2021). The aim of the Ivashkevych E’s study was to define different types of methods to test comprehension of the audio text heard, such as non-verbal means, verbal means and representational means (Ivashkevych and Komarnitska, 2020). Scheetz A M’s intent is that, based on exchange theory and the generalized reciprocity norm, employees’ perceived psychological contracts are believed to have dysfunctional consequences for the organization if violated (Scheetz and Fogarty, 2019). However, the depth of reading psychology still needs to be further refined and explored.

The emerging field of affective computing focuses on enhancing the ability of computers to understand and appropriately respond to people’s emotional states in human-computer interaction, and showing great potential for wide-ranging applications. Xin reviewed ten theoretical and operational challenges that existing affective computing research faced from an interdisciplinary perspective of information technology, psychology, and neuroscience. It is expected to help deepen the understanding of EEG-based affective computing and facilitate further applications (Hu et al., 2019). Hupont I first proposed a method in 2010. It is used to scalably fuse emotion channels in a 2D continuous emotion space, including a novel “emotional kinematics” filtering technique (Hupont et al., 2021). Htay M M proposed a feature extraction and classification method for FER. Facial expressions are an important role in affective computing and one of the non-verbal communication methods for human-computer interaction (Htay, 2021). The purpose of Tikhomirova D V is to study the dynamics of human emotional states during social interaction in virtual environments. The obtained results confirmed the eBICA model, showing its further extension and refinement (Tikhomirova et al., 2020). Hsu W C compared the use of non-hybrid BBL, hybrid BBL and non-BBL in three parts of the CS introductory course to determine whether any type of BBL is used to learn Java (Hsu and Gainsburg, 2021). Although the accuracy of these algorithms has reached a certain height, the performance of the algorithms needs to be improved.

Through the statistical analysis of the top 300 videos played by the Z Video software during the period from April 1, 2022 to June 1, 2022, the original content of the Z Video platform can be divided into three categories: editing original videos, filming Guest original and Paike + editor original. The innovation of this paper is that the psychological mechanism of reading is combined with affective computing. The theory and related methods of conditional random fields are introduced in detail, and the recurrent neural network is also introduced accordingly.

## Affective computing model approach

### Introduction to affective computing

In 1995, it was proposed that affective computing refers to the calculation of emotion-related factors caused by emotions or factors that may affect emotions. Emotional computing can perceive the degree of emotional performance of the party through some information and various sensors. The information can be verbal (such as music, chat voice, etc.), or physical (such as facial expressions, heartbeat, pulse, etc.), or just a simple text. Whether it is language or physiological signals to infer the user’s emotional state, the accuracy of judging emotions will be relatively high when most of the state is in contact with the user’s on-site feelings. But nowadays, with the development of the Internet, most netizens are accustomed to express their emotions on the Internet directly with words. Although it is not as accurate as the on-the-spot feeling, it is more flexible, convenient and practical to identifying the emotions of the parties through the text (Cambria et al., 2019; Yadegaridehkordi et al., 2019).

Emotional computing technology for text data can be divided into research tasks such as emotional object recognition, emotional information classification, and emotional reasoning. Different research tasks will use different algorithm models. Next, common models such as conditional random fields and loops neural networks will be introduced from the perspective of basic principles.

### Conditional random fields

Conditional random field (CRF) was proposed in 2001, which combined the characteristics of maximum direct model and hidden Markov model. The conditional random field is a conditional probability distribution model P(N| M), which represents a random Markov field. It contains a set of A random input variable M and another set of random output variables N. CRF is characterized by the assumption that the outputs form a random Markov field. Conditional random fields can be viewed as an extension of the maximum direct Markov model to the Markov salience problem. Peaks in a conditional random field graph model represent connections between random variable vertices, representing relationships between random variables. Conditional random field is a typical discriminant model. Its public probability can be written in the multiplicative form of many potential functions, of which the most commonly used is the random field under linear chain conditions (Jimenez-Zafra et al., 2019; Noroozi et al., 2019).

Random condition fields are often used in lexical analysis, such as Chinese word segmentation and part-of-speech tagging. In order to make the classifier perform Chinese word segmentation better, when labeling the data, the labeling information of the adjacent data should be fully considered. Normal classifiers struggle to do this, CRF excels at it. The purpose of part-of-speech highlighting is to give the part of speech (such as noun, verb, surname, etc.) for each word in a sentence. The part-of-speech of these words is often related to the part-of-speech environment of the word. Therefore, CRF can also be used for processing (Schuller, 2019).

The conditional random field model belongs to the undirected graph model, and the structure of the graph is free. The following takes the linear chain CRF as an example to introduce the related theory of conditional random fields, including the main idea, parameter evaluation and reasoning process.

#### Model description

**FIGURE 1:**
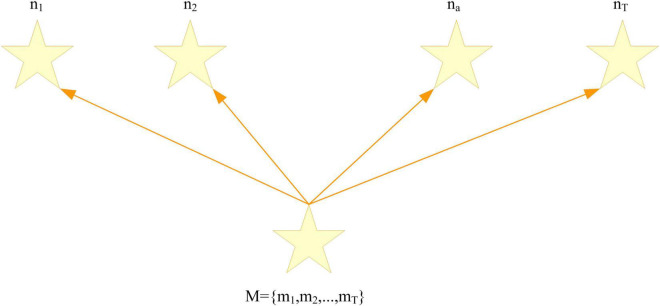
Linear chain conditional random field.

A random conditional field is a random Markov field that extracts a random variable N given a random input variable M, as shown in Figure 1. In a general random linear CRF, it is known that the input observation sequence *M* = {*m*_1_, *m*_2_, …, *m*_*T*_}, M is a random vector, the vector *m*_*a*_ represents the element at the a-th position, and there are T elements in total. The output marker sequence is *N* = {*n*_1_, *n*_2_, …, *n*_*T*_}. There are T elements in total and *n*_*a*_ represents the output tag corresponding to position *m*_*a*_, and *n*_*a*_ ∈ *S* represents a finite set.

Given input observation sequence X (value x), the conditional probability of output random variable Y (value y) is shown in Formula 1.


(1) $$p\,\left(n|m\right)=\frac{1}{Z\,\left(m\right)}\,\exp\left(\sum_{j=1}^{J}\kappa_{j}\,F_{j}\,\left(m,n\right)\right)$$

(2) $$Z\,\left(m\right)=\sum_{n}\exp\left(\sum_{j=1}^{J}\kappa_{j}\,F_{j}\,\left(m,n\right)\right)$$

(3) $$F_{j}\,\left(m,n\right)=\sum_{a=1}^{y}f_{j}\,\left(n_{a},n_{b},m,a\right)$$

Among them, J represents the total number of parameters, Z(m) is the normalization factor, φ = {κ_1_, κ_2_, …, κ_*J*_} is the parameter vector of the model, *f*_*J*_(*n*_*a*_, *n*_*b*_, *m*, *a*) is the feature function of the a-th position in the sequence. The function includes point features and edge features, and the point feature only needs to consider the point at the corresponding position a. The edge feature needs to consider not only the point a at the corresponding position but also another point b connected to the edge, as shown in the following formula.


(4) $$f_{j}\,\left(n_{a},n_{b},m,a\right)=\left\{ \begin{array}{c} f_{j}\,\left(m_{a},n_{a}\right)\\ f_{j}\,\left(m_{a},n_{a},m_{b},n_{b}\right) \end{array}\right.\,\begin{array}{c} \left(j\in J^{y}\right)\\ \;(a\neq b,j\in J^{y}) \end{array}$$

Among them, *J*^*y*^ represents the point feature set, *J*^*e*^ represents the edge feature set. The value is 1 when the function satisfies the feature condition, and the value is 0 when the condition is not satisfied.

#### Parameter estimation

Assuming that the training data set $X={\left\{m^{\left(a\right)},n^{\left(a\right)}\right\}}_{a=1}^{Y}$ contains Y sample data, each sample is an input sequence $m^{\left(a\right)}=\left\{m_{1}^{\left(a\right)},n_{2}^{\left(a\right)},\ldots ,m_{T}^{\left(a\right)}\right\}$ of length T, corresponding to an output sequence $n^{\left(a\right)}=\left\{n_{1}^{\left(a\right)},n_{2}^{\left(a\right)},\ldots ,n_{T}^{\left(a\right)}\right\}$ of length T. Parameter φ = {κ_*J*_} is estimated using maximum likelihood estimation, and the log-likelihood form is as follows:


(5) $$\max_{\varphi }\left(\varphi \right)=\sum_{a=1}^{Y}\log p\left(n^{\left(a\right)},|m^{\left(a\right)}\right)$$

In order to prevent over-learning, a Gaussian penalty term is introduced in the parameter estimation process to assist the parameter estimation. Therefore, the causal likelihood estimation of the linear chain conditional random field is expressed as follows:


(6) $$k\,\left(\varphi \right)=\sum_{a=1}^{Y}\sum_{t=1}^{T}\sum_{j=1}^{J}\kappa_{j}\,f_{j}\,\left(n_{t}^{\left(a\right)},n_{t-1}^{\left(a\right)},m_{t}^{\left(a\right)}\right)$$

$$-\sum_{a=1}^{Y}\log Z\,\left(m^{\left(a\right)}\right)-\frac{1}{2\,\varphi^{2}}\,\sum_{j=1}^{J}\kappa_{j}^{2}$$

Among them, *k*(φ) usually cannot directly find the maximum value, but it can be obtained by partial derivative.


(7) $$\frac{\partial k}{\partial \kappa_{j}}=\sum_{a=1}^{Y}\sum_{t=1}^{T}f_{j}\,\left(n_{t}^{\left(a\right)}\,n_{t-1}^{\left(a\right)},m_{t}^{\left(a\right)}\right)$$

$$-\sum_{a=1}^{Y}\sum_{j=1}^{J}\sum_{n,n^{\prime }}f_{j}\,\left(n,n^{\prime },m_{t}^{\left(a\right)}\right)\,p\,\left(n,n^{\prime }|m^{\left(a\right)}\right)-\sum_{j=1}^{J}\frac{\kappa_{j}}{\varepsilon^{2}}$$

The likelihood function takes its maximum value when the gradient is 0. Therefore, the optimization method of iterative approximation can be used to solve the parameters. Due to the limitation of a large number of parameters of the conditional random field model, the L-BFGS quasi-Newton method is usually used to calculate the model parameter eight.

#### Reasoning process

To reason about a conditional random field is to compute its probability. The reasoning process of the linear chain conditional random field model is similar to that of the hidden Markov model. It uses a forward-backward algorithm to calculate the probability recursively in a recursive way (Andre, 2019; Cui and Nan, 2019).

**FIGURE 2:**
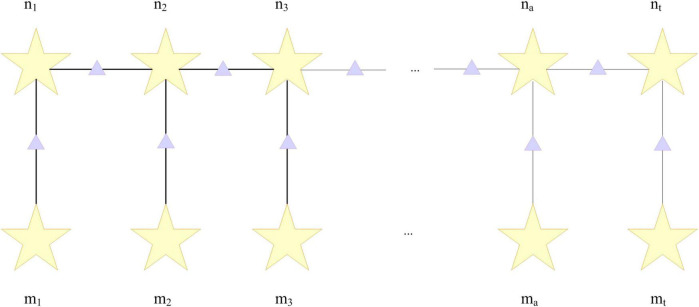
Linear chain conditional random field factor plot.

The conditional random field model is an undirected graph model. The linear chain conditional random field in Figure 1 is represented as a factor graph in Figure 2. The purple triangles in the figure represent factors, and the factors are represented as follows:


(8) $$\vartheta_{j}\left(a,b,m\right)\overset{d\,e\,f}{=}p\left(n_{a}=b|n_{t-1}\right)p\left(m_{a}=m|n_{t}=b\right)$$

Similar to the forward algorithm in the hidden Markov hidden model, this factor ϑ_*j*_(*a*, *b*, *m*) is given by the observation sequence μ. Therefore, the summary probability p(m) can be written as:


(9) $$\begin{array}{l} p(m)=\sum_{n}p(m,n)=\sum_{n}\prod_{t=1}^{T}\vartheta_{t}(n_{t},n_{t-1},m_{t})\\ =\sum_{nT}\sum_{nT-1}\vartheta_{T}(n_{T},n_{T-1},m_{T})\sum_{nT-2}\vartheta_{T-1}(n_{T-1},n_{T-2},m_{T-1})\sum_{nT-3}\ldots \end{array}$$

In the forward-backward algorithm, forward variables β_*t*_ can be pre-defined. The number of variables is the prediction length plus 1, that is, T + 1. Each variable is a vector of length *n*_*t*_. A vector with the number Q of values is used to save the intermediate summary results of the algorithm. The summary results are fixed and do not need to be involved in the calculation every time. The variable is defined as:


(10) $$\beta_{t}\left(b\right)\overset{d\,e\,f}{=}p\left(m_{<1\,\ldots \,t>},n_{t}=b\right)$$

$$=\sum_{n\,<1\,\ldots \,t-1>}\varepsilon_{t}\,\left(b,n_{t-1},m_{t}\right)\,\prod_{t^{\prime }=1}^{t-1}\varepsilon_{t^{\prime }}\,\left(b,n_{t^{\prime }-1},m_{t^{\prime }}\right)$$

The above formula counts the probability of outputting the random variable sequence *n*_<1,2,…,*t*–1>_ from the starting position to the t-1 position. The Formula 11 calculates the value of β_*t*_(*b*) in an iterative manner. Among them, the initial value is $\beta_{t}\,\left(b\right)=\varepsilon_{1}\,\left(b,n_{0},m_{t^{\prime }}\right)$


(11) $$\beta_{t}\,\left(b\right)=\sum_{a\in S}\varepsilon_{1}\,\left(b,a,m_{t}\right)\,\beta_{t-1}\,\left(b\right)$$

The backward iteration process is similar to the forward iteration, but in the opposite direction. The backward variable and iterative formula are as follows, and the initial value is β_*T*_(*a*) = 1, *p*(*m*) = α_0_(*n*_0_) = ∑_*n*1_ ε_1_(*n*_1_, *n*_0_, *m*_1_)α_1_(*n*_1_).


(12) $$\alpha_{a}\left(a\right)\overset{d\,e\,f}{=}p\left(n_{<t+1\,\ldots \,T>}|n_{t}=a\right)$$

$$=\sum_{n\,<t+1\,\ldots \,T>}\prod_{t^{\prime }=t+1}^{T}\varepsilon_{t^{\prime }}\,\left(n_{t^{\prime }},n_{t^{\prime }-1},m_{t^{\prime }}\right)$$

(13) $$\alpha_{j}\,\left(a\right)=\sum_{b\in S}\varepsilon_{t+1}\,\left(b,a,m_{t+1}\right)\,\beta_{t+1}$$

In a factor graph such as *m*_*t*–1_ and *m*_*t*_ given that *m*_<1,2,…,*t*–1>_ and *m*_<*t*+1,…,*T*>_ are independent of each other, then *p*(*n*_*t*+1_, *n*_*t*_|*m*) can be factored as:


(14) $$p\,\left(n_{t-1},n_{t}|m\right)=\varepsilon_{t}\,\left(n_{t},n_{t-1},m_{t}\right)$$

$$\left(\sum_{n\,<1\,\ldots \,t-2>}\prod_{t^{\prime }=1}^{t-1}\varepsilon_{t^{\prime }}\,\left(n_{t^{\prime }},n_{t^{\prime }-1},m_{t^{\prime }}\right)\right)$$

$$\left(\sum_{n<t+1,\ldots ,T,>}\prod_{t^{\prime }=t+1}^{t-1}\varepsilon_{t^{\prime }}\,\left(n_{t^{\prime }},n_{t^{\prime }-1},m_{t^{\prime }}\right)\right)$$

It can be obtained by iterative calculation of forward and backward through the following formula.


(15) $$p\,\left(n_{t-1},n_{t}|m\right)=\beta_{t-1}\,\left(n_{t-1}\right)\,\varepsilon_{t}\,\left(n_{t},n_{t-1},m_{t}\right)\,\alpha_{t}\,\left(n_{t}\right)$$

#### Prediction algorithm

In the case of inputting sequence and p(N| M), the conditional random field model predicts that when the conditional probability *n*^∗^ is greater, this is the sequence of labels corresponding to the expected sequence of observations. The prediction process of the conditional random field model is the same as that of the Markov hidden model. The calculation adopts the Viterbi dynamic programming algorithm (Oh et al., 2020). The prediction objective function is expressed as follows:


(16) $$\begin{array}{c} n^{*}=\arg\,\max_{n}p\,\left(n|m\right)\\ \begin{array}{cc} & = \end{array}\,\arg\,\max_{n}\frac{\exp\left(\sum_{j=1}^{J}\kappa_{j}\,F_{j}\,\left(m,n\right)\right)}{Z\,\left(m\right)}\\ \begin{array}{cc} & = \end{array}\,\arg\,\max_{n}\exp\left(\sum_{j=1}^{J}\kappa_{j}\,F_{j}\,\left(m,n\right)\right)\\ \begin{array}{cc} & = \end{array}\,\arg\,\max_{n}\exp\,\sum_{j=1}^{J}\kappa_{j}\,F_{j}\,\left(m,n\right)\; \end{array}$$

Therefore, the prediction problem is transformed into an optimization problem, which is to find the maximum value of the denormalized probability:


(17) $$\max_{n}\sum_{j=1}^{J}\kappa_{j}\,F_{j}\,\left(m,n\right)$$

### Recurrent neural network

**FIGURE 3:**
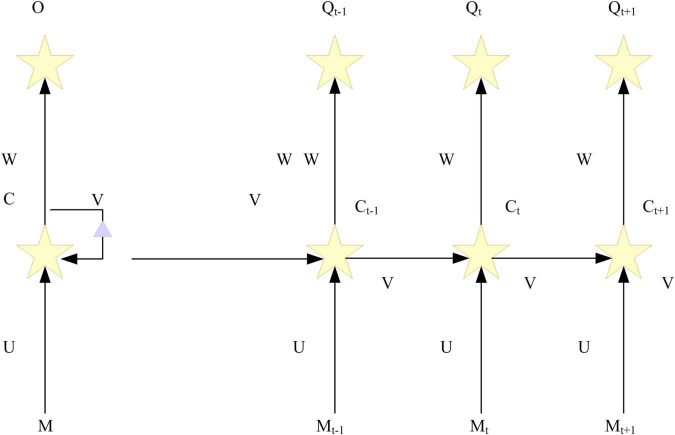
Recurrent neural network (RNN) model.

Neural network is an artificial neural network in which nodes are connected to a ring. The internal state of such a network can exhibit dynamic timing behavior. Neural network includes input, hidden, and output layers. The output is controlled by an activation function, and the layers are connected by weights. The activation function is determined in advance, and what the neural network model “learns” through training is included in the “weights”. Ordinary neural networks are independent of the environment and cannot solve problems that require contextual understanding. However, repetitive neural networks can combine contextual information to draw conclusions. RNN is a neural network that models sequential data (i.e., sequence), the current output is also related to the previous output. What RNN means is that the network will memorize previous information and incorporate it into the computation of the next output (Andre, 2020; Melo et al., 2021). Nodes between hidden layers are connected, not disconnected. The input of the hidden layer includes the input level and output of the hidden layer at the previous moment. Figure 3 is an example diagram of the RNN model:

Among them, *p*_*t*_ is the input at time t, *c*_*t*_ is the hidden state at time t. It is composed of the hidden state *c*_*t–1*_ at time t-1 and the input of current time *p*_*t*_, which expressed as *p*_*t*_ = *f*(*Up*_*t*_ + *Vc*_*t*−1_). The f in the formula is generally false linear activation function, which sets *c*_*–1*_ of the initial state to 0. *q*_*t*_ is the output at time t, and the vector of the next word can be represented by *q*_*t*_ = *soft max*(*Ws*_*t*_). Each layer in the RNN will share the same parameters (U, W, V in the above figure), which also means that each step in the RNN is performing the same operation. Using the same parameters for different inputs can effectively reduce the parameters that need to be learned in the network.

Recurrent neural network parameters are trained using BPTT (back-propagation through time algorithm), which is mainly used to train repeated layers (Ildiko, 2019). Its basic principle is the same as that of BP algorithm. It consists of three steps: (1) The forward calculation of each neural network. (2) The reverse value of the error term of each neuron, which is the neuron j weight the partial derivative of the error function E at the input. (3) Computing the gradient of each weight. Finally update the weights using the stochastic gradient descent algorithm.

The input of traditional neural networks and convolutional neural networks is a fixed vector, the output is also a fixed vector. The number of layers in the model and the steps performed by the algorithm are also fixed. RNN is a neural network model with memory ability, which can store environmental information in text, and its input and output can be sequence data (An et al., 2020; Nazarov, 2020). Supposing there is an input sequence that is *P* = {*p*_1_, *p*_2_, …, *p*_*T*_}, it can be updated as following formula.


(18) $$k^{\left(t\right)}=f\,\left(U\,p^{\left(t\right)}+V\,p^{\left(t-1\right)}+j\right)$$

**FIGURE 4:**
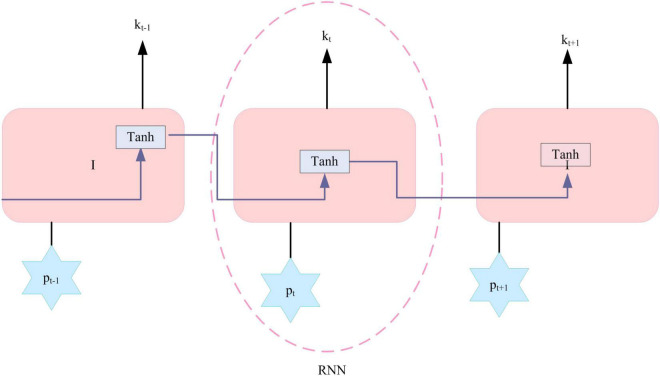
Network structure of a standard recurrent neural network (RNN).

Among them, f is the non-linear activation function mentioned above, U, *V* ∈ *R^Hxd^*, *j* ∈ *R*^*H*^ are the parameters to be trained by the model. *k*^(*t*)^ ∈ *R*^*H*^ is the vector belonging to the hidden layer and is a memory unit of the network. j is the bias term, and d represents the dimension of the input layer, K represents the dimension of the hidden layer, as shown in Figure 4.

## Experiments of news communication strategies

### Production and broadcasting of news content

This paper selected the 300 most-played news short videos released by Z software in the period from April 1, 2022 to June 1, 2022 as research samples, and encoded them. A total of 300 news short videos were encoded, and multi-dimensional content analysis was performed on them.

#### Content source

Sustainable content production capacity is the first consideration for content entrepreneurs. The current content production mode of Z Video Software is a news content aggregation mode jointly produced by platform originals and self-media.

**FIGURE 5:**
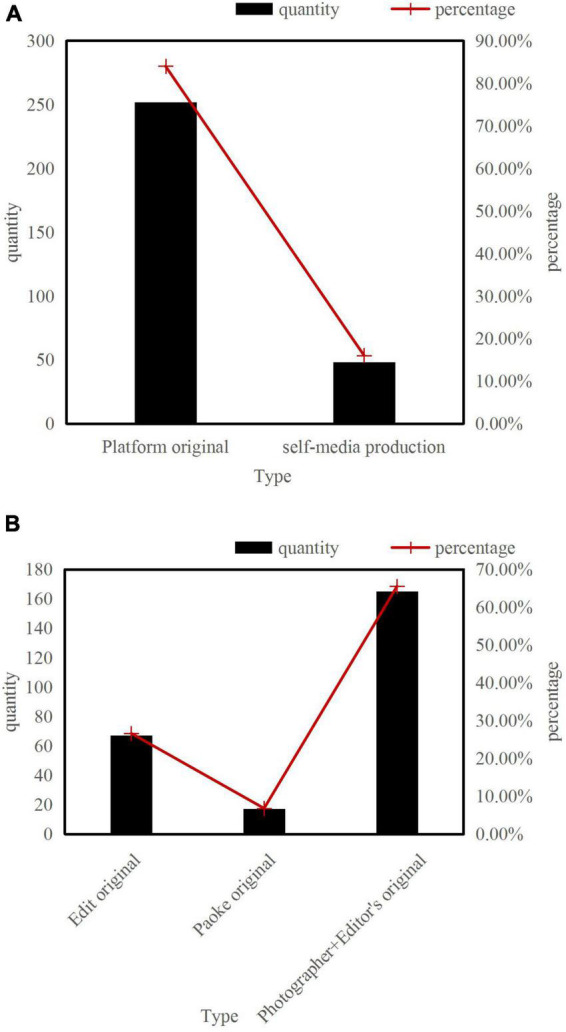
Video content composition and material source of Z Video news information. **(A)** Composition of Z Video news and information video content. **(B)** Sources of Z Video news and information video content.

According to the statistical analysis of the top 300 videos played by Z Video software during the period from April 1, 2022 to June 1, 2022, 252 videos are original content of Z Video software, accounting for 84.00%. Other short videos come from the platform’s self-media, as shown in Figure 5A.

The original content of the Z Video platform can be divided into three categories: one is editing original videos, including short videos that Z Video independently collects, edits and distributes, and short videos that edit and process the original materials of TV stations, radio, and other media. The second is the original content of Paike, that is, users who have obtained the qualification of Paike shoot and complete the original content that is edited and submitted to Z Video. The third is Paike + editor original. The editor determines the topic, and the photographers who are distributed in the world of Z Video upload news according to their needs. The content is edited by professional editors after screening. Among the 252 original short videos on the platform, a total of 165 videos from Paike + editing accounted for 65.48%, 67 original videos for editing accounted for 26.59%, and 17 original videos for Paike accounted for 6.75%, as shown in Figure 5B.

(a) Composition of Z Video News and Information Video Content.
(b) Sources of Z Video News and Information Video Content.

At present, Z Video can produce more than 600 news short videos every day, which can achieve large-scale content production. It is far from enough to rely on Z ‘Video’s own employees. To a large extent, it benefits from the system of shooting customers which has established around the world. Currently, Z Video has more than 10,000 key photographers and more than three°million photographers in hundreds of cities around the world. For Z Video, Paike is a source of living water for video content. Compared with the previous traditional media production mode, the advantage is that the cost is relatively low. The same cost can use more resources and a larger human network. In this way, it can improve the response of information. It can also improve the richness and diversity of information content. The UGC content produced by Paike is basically sufficient in the Z Video content pool, but the disadvantage is that most Paokes have not received professional journalism education.

#### Classification of content

The current columns of Z Video are divided into 14 columns: new knowledge, society, world, life, technology, entertainment, wealth, automobile, food, sports, music, two-dimensional, funny, as shown in Table 1. Through the analysis of 300 news short videos of Z Video, social life news (including three columns of society, world, and life) accounts for up to 54.00%. The current affairs breaking news is less involved, and most of the content focuses on the life and fate of the little people in the context of the big era and other content with a strong firework atmosphere. The information that Z Video understands is more about defining news as hot topics on social platforms, and many of these topics have little relevance to current political news in the traditional sense.

**TABLE 1:** Topic distribution of Z Video news and information videos.

	Quantity	Percentage
Society	104	34.67%
World	30	10.00%
LIFE	28	9.33%
Entertainment	23	7.67%
New knowledge	23	7.67%
Food	17	5.67%
Funny	16	5.33%
Music	10	3.33%
Wealth	14	4.67%
Technology	13	4.33%
Two-dimensional	7	2.33%
Physical education	6	2.00%
Car	9	3.00%

Through the research of previous literature and the reading of major data news columns, the topics of data news are roughly divided into eight categories: international, political, economic, cultural, social, environmental, popular science, and others (including sports, entertainment, etc.). After sorting out all the data news works of Netease’s “Digital Reading”, People Daily’ “Illustrated News”, and Caixin’s “Numerical Talk” for the whole year of 2021, the results are as follows:

(a) Distribution of Topics Selected by the Caixin Client “Digital Talk” and the People’s Daily Online Graphic News Official Account.
(b) Distribution of Topics Selected by NetEase Digital Reading Weibo, Official Account, and Client.

**FIGURE 6:**
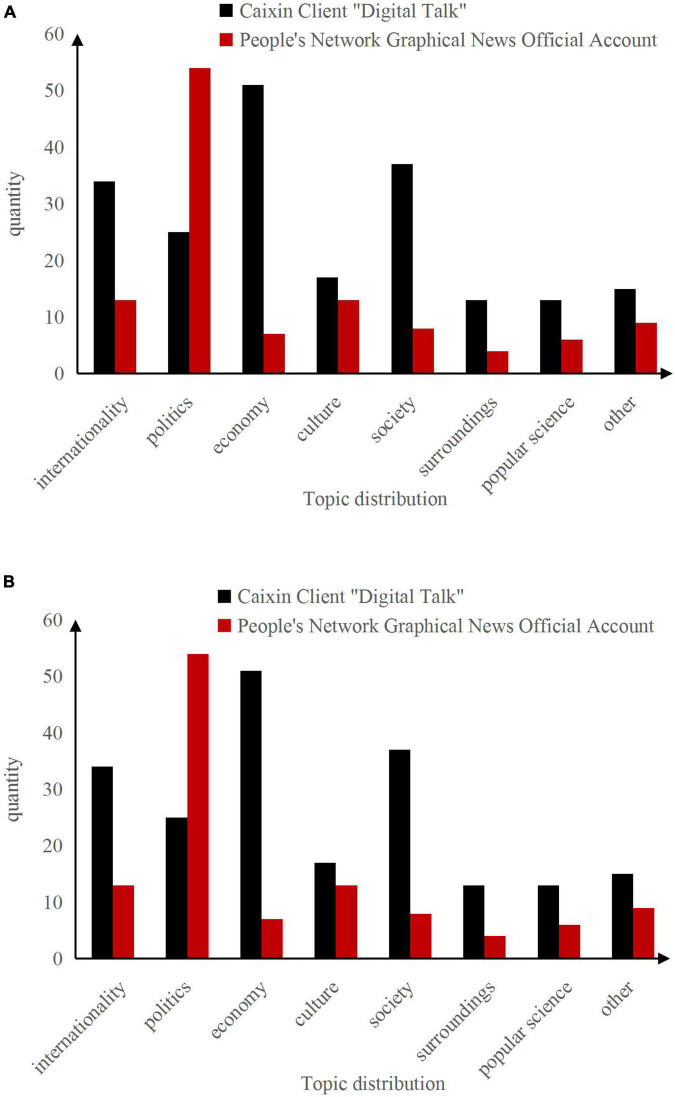
Topic distribution. **(A)** Distribution of topics selected by the Caixin client “Digital Talk” and the people’s daily online graphic news official account. **(B)** Distribution of topics selected by NetEase digital reading Weibo, official account and client.

The statistics of Caixin’s mobile news client “Digital Talk” in Figure 6A showed that the distribution of topics includes 34 articles in the international category, 25 articles in the political category, 51 articles in the economic category, 17 articles in the cultural category, and 37 articles in the social category, 13 articles in the environmental category and popular science category, and 15 other articles. Statistics showed that financial topics account for about 24.88%, which is the most frequently reported among all topics. This topic mainly includes varieties of financial topics. Taking China’s financial reports as an example, the topic is selected generally in real estate, finance, corporate comparison, national policies, etc. International financial reports cover international import and export trade, international finance, world monetary policy, and other aspects. Compared with other data news columns, the topic selection of “Digital Talk” is more inclined to the economic and financial fields, which is very consistent with Caixin’s positioning of disseminating economic and financial content.

The statistical results of the distribution of topics on NetEase’s Weibo account in Figure 6B show that the distribution of topics includes three papers in the international category, one article in the political category, seven articles in the economic category, 13 articles in the cultural category, 39 articles in the social category, and three articles in the environmental category, four articles for popular science, and eight articles for other categories. From this, it can be seen that the original data news topics of NetEase’s “Shu Du” are more inclined to the social and cultural fields. These two topics account for 66.67% of the total selected topics. It can be seen that the topics selection of cultural news reports in “Shu Du” are very diverse, including regional customs, music culture, ancient and modern history, food exploration, differences between North and South, etc., such as *How much do Chinese people love to eat hot pot*? “*China’s obesity map is released, and the waist of northerners is thicker*. These reports allow readers to have a new understanding of current Chinese culture in all aspects through the collection, analysis, and summary of data. The topic selection is not timely and news, but it is full of interest.

Figure 6B shows the distribution of topics for different new media platforms of NetEase’s “Shu Du”. Unlike Weibo, the number of articles published on the official account is more than that on the client and Weibo, while the number of articles published on the client is the lowest. The overall topic selection is similar to Weibo, and it is mostly concentrated in social and cultural categories.

#### Processing of content

From the perspective of video duration, the duration of short news videos released by Z Video is mainly concentrated in the range of 20 s to 2 min, accounting for 78.67% of the total. Among them, videos with a length of 0–1 min accounted for 45.00% at most, as shown in Table 2.

**TABLE 2:** Z Video news video duration.

	Quantity	Percentage
0–1 min	135	45.00%
1–2 min	101	33.67%
2–3 min	33	11.00%
4–5 min	18	6.00%
More than 5 min	13	4.33%

Through the analysis of the duration of news short videos released by Z Video, it is believed that the current duration of news short videos is basically less than 2 min. The “conciseness” of news clips is mainly reflected in the following aspects. First, selecting a single reference angle. Cutting from the most interesting direction of the event and focus on just one direction in the coverage. For example, when reporting on the theme of the Spring Festival, the stories of specific people affected by the Spring Festival are often chosen to reflect the general context at the time. Second, minimizing unnecessary reporting details. Short news information videos do not need to adhere to the editing concept of traditional TV news. It tend to reduce auxiliary shots and close-ups as much as possible to express feelings only. Furthermore, go straight to the point and make it clear. News short videos do not need to meet the reporting framework of traditional news, such as adding pavement and opening. Instead, it is to strengthen and edit the most exciting and conflicting parts of a piece of news. Users want to make a decision whether to continue watching immediately after clicking on the video, so it should be presented faster and more directly.

### Communication strategy of integrated news

**FIGURE 7:**
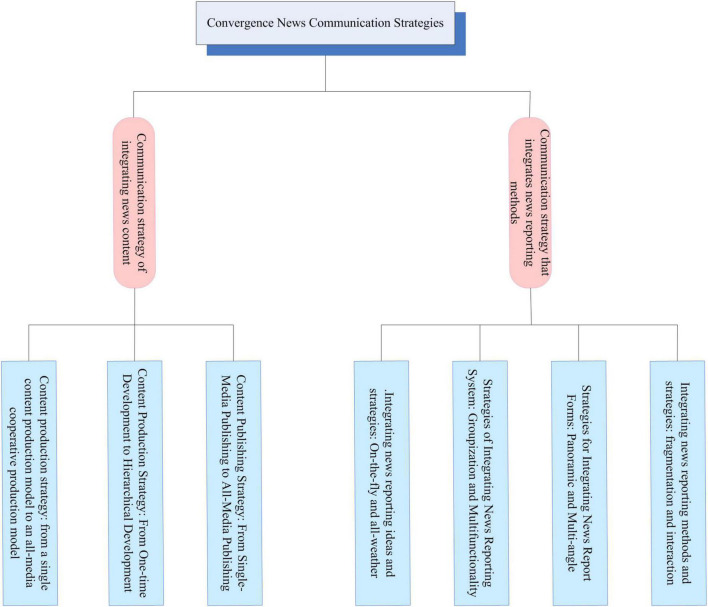
Communication strategies for convergent news.

In the era of traditional media, the traditional news dissemination form is relatively single, the sources of information content are limited, the production mode of linear operation, the one-way communication channel, the poor interaction of the audience, and the excessive separation of content sections. These defects make the traditional news industry face severe challenges. In order to adapt to the development trend of communication technology and the audience’s higher requirements for information, traditional media have begun to integrate with each other, and simple mechanical copying and content transplantation have been carried out. However, this has not fundamentally changed the “point-to-face” of traditional media. The way of information transmission and reception just enriches the presentation of information. Subsequently, with the rapid development of modern information technology with digital technology as the core, the media industry has begun to evolve from the traditional media era to the new media era. The development of digital technology can break through the morphological barriers of different media, and promote the formation of deep-level media in terms of content, technology and terminals. The fusion is no longer a simple addition in form. The emergence of fusion news is the product of the internalization of this media fusion concept into practice. On the basis of inheriting traditional news, it innovated the content and reporting methods of traditional news, and formed some new communication strategies. It is mainly reflected in the following aspects, as shown in Figure 7.

The content view of traditional media is mainly reflected in a static content view in the context of a single media industry. The single content system and linear communication method make the production, integration and application of content unable to meet the diverse needs of the audience. The form of expression is also developing steadily, and it cannot extend the connotation and value of the product. In the new media era, content is still the core and pillar of the media industry. The integration function is an important way for media to improve their own communication capabilities. It broken the original inherent boundaries of various media, and there are many innovations in content production, production and distribution, which reflected the revolutionary changes brought by the trend of media integration to the practice of news communication. Therefore, it is of positive significance to explore a practical content integration model for the optimization and development of the media.

The way of news reporting is a dynamic expression of media dissemination of information. In the age of traditional media, traditional news reporting is limited by the limited technical resources and can only implement a single content reporting method in line with its own media attributes. To a certain extent, the multiple selectivity of the audience to obtain information is limited, and the frequency of interaction between the two parties in the dissemination process is also weak. The disseminator cannot obtain the feedback and opinions of the audience in time, resulting in low efficiency of information dissemination. With the development of fast-paced life at this stage, especially in the information age, the audience’s intention to pursue personalized information and master the dominance is getting stronger and stronger. The reporting methods and dissemination forms of news in the traditional media era are far from meeting these basic needs of the audience. The fusion news generated based on the background of media fusion can combine images, text, sound, video, animation, and other elements freely. It has formed a variety of products such as electronic newspapers, digital broadcasting, digital TV, online news, mobile phone newspapers, etc. The news reporting methods are rich and diverse, and it pays great attention to the deep interaction with the audience. The audience can freely choose the receiving form they need and are interested in. The communicator can also understand the audience’s cognition and feeling of the information in the first time through the network media. They can further improve the report according to the interests of the audience in time, so as to provide the audience with personalized and comprehensive information. This transformation of integration of news reporting methods has greatly improved the information dissemination capabilities of various media, expanded audience resources and helped to enhance the effect of content dissemination.

During the study period, more than 40% of the public had simultaneous exposure to more than five types of media; nearly 60% of the public had simultaneous exposure to television, radio, and the Internet; and almost 70% of the public had simultaneous exposure to the Internet, car TV, and newspapers. It can be seen that in the era of media convergence, the user market is increasingly subdivided. Table 3 shows the exposure ratios of different forms of audience to different media.

**TABLE 3:** Proportion of audiences with different forms of exposure to different media.

Media	Modern open	Follow suit	Advertising alienation	Moderate casual	Fusion adaptive	Career striving
Television	98.93	99.11	99.20	98.96	99.12	99.24
Broadcast	41.55	33.73	23.03	23.06	45.84	38.79
Network	67.32	39.64	31.26	31.54	51.66	58.68
Newspaper	71.35	64.32	42.82	49.82	89.03	78.14
Magazine	53.18	35.85	24.95	29.13	52.04	50.81
Movie	24.62	11.42	8.52	8.84	16.83	21.98
Mobile TV	3.14	1.78	1.37	1.71	2.01	3.13
Car TV	53.39	43.22	39.13	36.25	54.33	58.76
Building outdoor	51.23	37.29	31.48	31.10	49.13	53.93

As can be seen from Table 3, different groups and different types of audiences have different preferences for media exposure. Therefore, in the production and development process of convergent news, this characteristic change of the audience should be firmly grasped and the target audience’s psychology should be clarified. Then making developed strategies for focusing and disseminating news creative news products. The personalized communication characteristics of new communication technologies have created an open and equal communication platform for the public, which allows the public to participate in the wide dissemination of information. At the same time, it encourages and motivates the public to actively produce communicative content and establish their own voice. Converged news developed by relying on new communication technologies should encourage audiences to actively participate in the content production process, strengthen interaction between the two parties and tap the real needs of audiences, so as to provide audiences with customized news products.

### Future trend of converged news development

**FIGURE 8:**
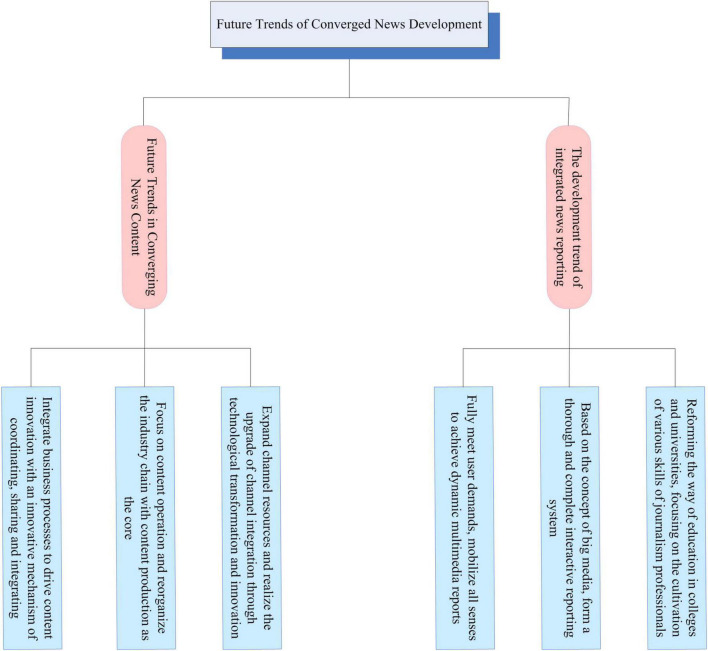
Future trends in convergent news development.

Converged news breaks through the limitations of traditional news in terms of communication subjects, operational concepts, production methods, communication processes, and organizational management. This new form based on the new identity change has brought a variety of communication effects. With the continuous development of digital technology, the strength and closeness of media integration will continue to increase. The methods to optimize the integration of news content and reporting methods also need to be further explored, as shown in Figure 8.

As a key element of media market competition, content resources have always been the key to all media competitions. Especially high-quality, original, authoritative, effective and scarce content resources can drive the rapid development of media and have unique characteristics in market competition. Therefore, comprehensive news should make full use of content resources, focus on individual users, and create a new content system of “multimedia production–full media release” in the future development.

With the continuous improvement of social productivity, people’s consumption choices and methods are gradually diversified. Only by continuously innovating products and enriching product functions can manufacturers win the attention and choice of the audience in the fierce market competition. The same is true in the field of media. In the 21st century, the progress of media technology has changed the way of news reporting. In the process of integration and mutual penetration of traditional media and new media, the way of news reporting has become more diversified, showing obvious characteristics of media integration. Converged news, as the core reporting concept of media integration, must also adapt to the situation in the future development, change its thinking and implement the optimal news reporting method.

## Conclusion

The development of technology and human needs have jointly promoted the evolution of media forms, giving birth to short video, a new medium form that conforms to the characteristics of Internet communication. Short videos have become an excellent carrier for news dissemination by virtue of their characteristics such as instant upload, small volume but rich content carrying capacity, and dynamic social language. The combination of news and short videos has produced a new form of news—short videos of news information. Its emergence has subverted the complex process of collecting, editing, producing, and disseminating of traditional news. These complex processes can be easily completed through the application of mobile terminals. In addition, the decentralization of the media’s discourse power and the transformation of the role of receivers to transmitters have not only improved the efficiency of news production but also ensured the continuous output of content. The video presentation makes the news form integrated with text, images, and sounds, making it more three-dimensional and vivid. Multi-channel and multi-terminal distribution breaks the barriers of news reception scenarios. Lightweight information carrying and straight-to-the-point content fill the needs of users for news information in the fragmented time. News short videos have become an important means of media reporting and the main development trend of information dissemination in the era of mobile communication.

## Data availability statement

The original contributions presented in this study are included in the article/supplementary material, further inquiries can be directed to the corresponding author.

## Author contributions

BX: writing – original draft preparation, editing data curation, and supervision.
